# Liquid Biopsies in the Clinical Management of Germ Cell Tumor Patients: State-of-the-Art and Future Directions

**DOI:** 10.3390/ijms22052654

**Published:** 2021-03-06

**Authors:** João Lobo, Ricardo Leão, Carmen Jerónimo, Rui Henrique

**Affiliations:** 1 Cancer Biology and Epigenetics Group, IPO Porto Research Center (GEBC CI-IPOP), Portuguese Oncology Institute of Porto (IPO Porto) & Porto Comprehensive Cancer Center (P.CCC), R. Dr. António Bernardino de Almeida, 4200-072 Porto, Portugal; jpedro.lobo@ipoporto.min-saude.pt; 2 Department of Pathology, Portuguese Oncology Institute of Porto (IPOP), R. Dr. António Bernardino de Almeida, 4200-072 Porto, Portugal; 3 Department of Pathology and Molecular Immunology, Institute of Biomedical Sciences Abel Salazar, University of Porto (ICBAS-UP), Rua Jorge Viterbo Ferreira 228, 4050-513 Porto, Portugal; 4 Faculty of Medicine, University of Coimbra, Rua Larga, 3000-370 Coimbra, Portugal; romaoleao@gmail.com

**Keywords:** germ cell tumors, liquid biopsies, microRNAs, DNA methylation, circulating tumor cells, biomarkers, diagnosis, follow-up, serum tumor markers

## Abstract

Liquid biopsies constitute a minimally invasive means of managing cancer patients, entailing early diagnosis, follow-up and prediction of response to therapy. Their use in the germ cell tumor field is invaluable since diagnostic tissue biopsies (which are invasive) are often not performed, and therefore only a presumptive diagnosis can be made, confirmed upon examination of the surgical specimen. Herein, we provide an overall review of the current liquid biopsy-based biomarkers of this disease, including the classical, routinely used serum tumor markers—the promising microRNAs rapidly approaching the introduction into clinical practice—but also cell-free DNA markers (including DNA methylation) and circulating tumor cells. Finally, and importantly, we also explore novel strategies and challenges for liquid biopsy markers and methodologies, providing a critical view of the future directions for liquid biopsy tests in this field, highlighting gaps and unanswered questions.

## 1. Introduction: Current Clinical Challenges in Germ Cell Tumors

Germ cell tumors (GCTs) represent a heterogeneous group of neoplasms that emerge due to disturbances in embryonic and germ cell development. For this reason, a developmental model of the disease has been the mainstay of these tumors’ classification, where each tumor subtype and category reflects the molecular background (including epigenetic) of the corresponding cell of origin [1]. GCTs may then arise in distinct age groups (from pediatrics to old adulthood) and in both genders, both in the gonads (testis and ovary) and also in extragonadal sites (due to impairment of migration of primordial germ cells along the midline of the body) [2]. Among these, GCTs of the testis (TGCTs), particularly the so-called type II TGCTs of the young-adult male, are the most common and well-studied, constituting the main focus of this review. These are derived from a precursor lesion (germ cell neoplasia in situ, GCNIS) and further divided in two major classes, the more homogeneous seminomas and the heterogeneous group of non-seminomas, which include several subtypes related to embryonal features (embryonal carcinoma), extra-embryonal features (yolk sac tumor and choriocarcinoma), somatic features (teratoma) and mixtures of any component (the mixed tumors) [3]. This categorization is clinically relevant since non-seminomas are frequently more aggressive, impacting on the appropriate treatment strategy [4].

**Figure 1:**
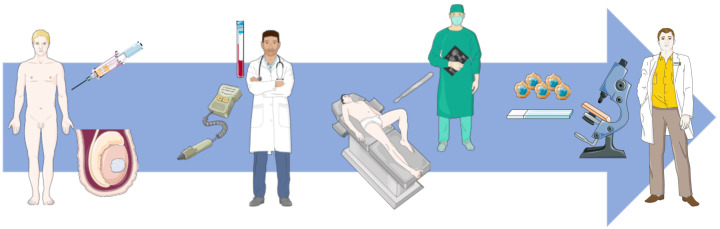
Clinical path of a patient with a testicular mass. Clinical work-up includes physical examination, scrotal ultrasound and classical serum tumor markers (AFP, HCG, and LDH) evaluation. The patient is ultimately submitted to removal of the testis (orchiectomy) and the definitive diagnosis (which can be a germ cell tumor, another tumor or even a benign inflammatory condition) is only provided by histopathological examination of the specimen.

TGCTs are the most common solid cancers in young adult Caucasian men. Their incidence is rising around the world, reported to be due to changes in lifestyle and early exposure to risk factors, some of which disturb the normal germ cell development in the developing embryo [5,6,7]. In most instances, TGCTs are diagnosed in their localized form, although 30% of patients already present with regional metastases or disseminated disease [2]. After noticing a palpable testicular mass, the patient generally refers to a physician, who orders a scrotal ultrasound, confirming evidence of a mass-forming lesion. Furthermore, so-called “classical” serum tumor markers (alpha fetoprotein (AFP), human chorionic gonadotropin (HCG), and lactate dehydrogenase (LDH)) are commonly ordered [8]. However, these are not robust enough to establish the diagnosis of a TGCT, being elevated in only 60% of patients at diagnosis and highly dependent on histology and stage [9]. While in other topographies a diagnostic tissue biopsy is mandatory to confirm the diagnosis, testicular biopsy is controversial and usually not performed, due to the (theoretical) associated risk of tumor cell seeding due to scrotal violation, with the procedure being performed in some countries for assessing the contralateral testis only [10]. The clinician is then left with a confirmed testicular mass (which may also correspond to a non-germ cell malignancy or even a benign condition) and a radical orchiectomy is needed for treatment and histopathological diagnosis. As such, accurate, non-invasive, means of diagnosis through liquid biopsy testing are an unmet need appreciated in this particular field (Figure 1).

Liquid biopsy is becoming more and more popular in patient management, particularly in oncology. Liquid biopsies allow to mimic tumor dynamics across time and to appreciate tumor heterogeneity when tissue sampling is difficult or non-representative of the whole tumor. The constant evolution in methodologies and techniques available for accurately determining and quantifying liquid biopsy biomarkers in several bodily fluids is prompting their introduction in the clinical context [11]. In TGCTs, this has been one of the most active niches in the field, with multiple research works aiming at finding the best biomarkers for covering all different clinical needs and complementing the current diagnostic and follow-up procedures [12]. Indeed, there are several clinical settings in need of better non-invasive liquid biopsy biomarkers. Besides the need to improve pre-orchiectomy diagnosis, better means of follow-up of these patients are needed that reflect response to therapy (both surgical and systemic) and accurately predict relapses in a timely manner, allowing less frequent routine imaging scans [13]. Risk stratifying/prognostic biomarkers are particularly relevant in the context of stage I disease, since there is a serious risk of overtreatment, with patients never destined to relapse ending up being treated with adjuvant cisplatin-based chemotherapy [14,15], which has associated side effects [16,17]. To date, such risk stratification has relied on clinical and histological parameters, such as lymphovascular invasion for non-seminomas and size and *rete testis* invasion for seminoma [18,19,20], leaving room for non-invasive markers to step in. Better ways of assessing post-chemotherapy residual disease are required, specifically to allow discrimination of necrosis/fibrosis and teratoma from viable non-teratoma GCT components, since the treatment strategy (including the decision to operate) could be adjusted if this information was known upfront [21].

In this manuscript, we provide an overall review of the panorama of liquid biopsies in GCTs. Here, we critically describe the role of the currently available serum tumor markers, discussing their use and unmet needs. While most studies on alternative biomarkers have interrogated/evaluated microRNAs (especially miR-371a-3p), we also provide an overview of studies on cell-free DNA (cfDNA) and circulating tumor cells (CTCs). Finally, we discuss the future applications of liquid biopsies in TGCTs.

## 2. Classical Serum Tumor Markers

The classical serum tumor markers AFP, HCG, and LDH are universally part of the management of patients presenting suspicious testicular lesions [22]. The elevation of such markers is suggestive of a TGCT and reflects disease burden, namely disease stage and tumor size [9], but these markers overall lack the necessary sensitivity (since elevations of AFP and HCG are mostly related to the presence of specific histological elements that may be present or not in yolk sac tumor and choriocarcinoma, respectively) and specificity (since all three can be elevated in other conditions) [23]. The fact that only 60% of patients present with elevations in these markers importantly leaves out 40% of patients with a testicular mass that cannot be reliably determined to be a (malignant) TGCT or another testicular condition, whether a tumor or not. This is particularly important in seminomas, which only infrequently (18 to 31%) lead to the elevation of serum tumor markers (HCG, since AFP is by definition negative) [9,24,25].

The link to developmental biology explains the use of both AFP and HCG as biomarkers of GCTs. The glycoprotein AFP, for instance, was first detected in high concentrations in human embryonal and fetal serum, at that time being named alpha-1 globulin [26]. It is, indeed, the major protein in serum of early mammalian embryos [27], being synthetized in the site of embryonal hematopoiesis, the yolk sac. Following this rationale, the work of Abelev et al. [28] documented for the first time that non-seminomas disclosed elevation of this serum marker, which later was shown to be, precisely, a marker of yolk sac tumor histology [29]. In fact, elevated AFP should trigger the pathologist to search for yolk sac tumor foci, easily overlooked admixed between other tumor components [3]. AFP elevation is documented in about 60% of non-seminomas [30]. Since hematopoiesis is, later in life, transferred to the liver, where fetal hematopoiesis takes place, and can also take place briefly in the gastrointestinal tract, it is not unexpected that around 20 to 25% of teratomas may have elevations of AFP (since these can have representations of intestinal-type glands and also fetal liver or hepatoid differentiation) [24]. There are two features worth noticing concerning the kinetics of AFP: the half-life of the marker in circulation is five to seven days, and the marker is expected to be elevated during the first year of life (levels decline progressively postnatally), with consequences in the interpretation of measurements in this age group, which can also have testicular masses, namely pediatric-type yolk sac tumors [31]. One of the limitations of AFP is related to elevations in other conditions, which include malignancies such as hepatocellular carcinoma and others, such as lung and pancreatic cancers [32,33] or the recently acknowledged gastric adenocarcinoma with enteroblastic differentiation [34], but also include other non-tumor conditions such as various forms of chronic liver disease, ataxia-telangiectasia and patients undergoing surgical procedures involving the gastrointestinal tract and liver [23]. In particular, GCT patients undergoing systemic treatments with cytotoxic drugs may show false-positive elevations of AFP, possibly due to liver injury, which can be challenging to interpret [35]. Moreover, hereditary elevations of AFP can be seen, which further complicates the laboratory workup of these patients presenting with testicular disease [36].

HCG, on the other hand, is a molecule with five different bioactive isoforms that has for a long time been the cornerstone of the commonly used pregnancy test. It is produced by placental cells (the syncytiotrophoblast cells) and plays various roles during pregnancy, fundamental for the mother, for the fetus and for reaching a fully developed placenta [37]. In fact, this hormone was shown to promote the differentiation of fetal organs [38]. Again, unsurprisingly, it has been proven to be a biomarker of trophoblastic disease, including choriocarcinoma (frequently with remarkably high serum levels) or syncytiotrophoblast cell foci variably present in GCTs (which elicit lower elevations of this marker, namely in 15 to 20% of pure seminomas) [39]. The half-life of HCG is 12 to 36 h. Similar to AFP, elevations of this marker should trigger the pathologist to procure such elements in the orchiectomy specimen. However, and also similar to AFP, HCG can be elevated in various settings, including in several malignancies [40] and due to heterophile antibodies [41]. Additionally, it is important to notice that elevated compensatory levels can be found in cases of hypogonadism (which may occur in patients previously submitted to orchiectomy) [42]. Although an association with marijuana consumption has been suggested, this has not been confirmed in larger studies [43].

The uses of LDH in the clinic are many, and the major characteristic and at the same time the drawback of this biomarker is that elevations can occur in many physiological and disease states, justifying its very low specificity [44]. The list of conditions that may present with elevated LDH is long and not limited to myocardial infarction, pulmonary embolism, hemolysis, skeletal muscle disease, among others. Overall, elevated LDH levels can indicate increased cell turnover (being routinely ordered in clinical care in the “general” blood work-up of hospital admitted patients), and are an indication of tumor lysis syndrome, which can happen in many malignancies [45]. Hence, it closely represents the bulk of disease. LDH is expressed ubiquitously (and very particularly in muscle) and has a role in the regulation of cell metabolism, catalyzing the conversion of lactate to pyruvate [46]. The LDH-1 isoform is the most expressed in TGCTs [47], and LDH is elevated in TGCTs in 40 to 60% of the cases, overall. The half-life of LDH is difficult to determine and its assay measures enzymatic activity, which can render substantial variability between assays from different laboratories [24].

Nevertheless, measurement of these markers is recommended both pre- and post-orchiectomy [22]. The former is important, for instance, as a baseline assessment of the disease burden and interpretation of subsequent levels post-orchiectomy and during follow-up. In certain instances, these markers can motivate a presumptive diagnosis and determine further actions (such as in the case of extremely high elevations in patients with a significant burden of disease including life-threatening metastatic dissemination, entailing the initiation of urgent treatment, or in the case of extragonadal tumors when biopsy risk is high [48]. Moreover, a relevant and sustained elevation of AFP in a tumor determined to be pure seminoma histologically dictates that, clinically, the patient should be considered a non-seminoma [49]. Post-orchiectomy measurements are mandatory, since they are part of the staging protocol (motivating a further category in the classical TNM staging (“S” stands for serum tumor markers) and should also be performed before initiating another chemotherapy cycle and at the end of systemic treatments, so as to monitor the patients’ response [22]. In fact, a persistent elevation of serum tumor markers that occurs during chemotherapy indicates the presence of a cisplatin-resistant phenotype, which is very challenging to treat since there is a lack of effective therapies for these patients, who eventually die from disease [50,51]. Measurements of classical serum tumor markers, together with routine check-ups and CT scans are frequently performed for follow-up of these patients (as mentioned, particularly important in stage I patients recommended for surveillance strategy), in order to detect possible recurrences early. Generally, 10 to 20% and 20 to 30% of seminoma and non-seminoma patients, respectively, present localized disease on surveillance relapse, the vast majority within the first two years of follow-up [52,53]. However, the fact is that the sensitivity of these classical markers for detecting relapses is rather low, especially in seminomas (only 3% of stage I seminomas were detected by elevation of serum markers) but also in non-seminomas (half of the relapses being marker-negative) [52]. Furthermore, the low specificity of LDH also renders this marker effective for detecting relapse [54]. Finally, in metastatic disease, post-orchiectomy levels [55] of serum tumor markers are determinant for establishing the corresponding International Germ Cell Cancer Collaborative Group (IGCCCG) category in non-seminomas (since seminomas can be attributed “good” or “intermediate” groups based solely on absence or presence of non-pulmonary visceral metastases), which in practical terms determines their prognosis (including survival) and the subsequent recommended therapy [56].

Some other serum markers including human placental alkaline phosphatase (PLAP), TRA-1-60 and neuron-specific enolase (NSE) have additionally been studied as putative serum biomarkers of TGCTs. However, and despite some good results in terms of sensitivity and representation of disease burden, false-positive elevations in frequent instances (such as elevations of PLAP in smokers) precluded their introduction in the clinic [57,58,59,60,61,62,63]. A recent study exploring specific N-glycans showed promising data in serum samples of 54 TGCT patients, providing an area under the curve of 0.87 in discriminating patients from controls, and also carried prognostic information, but needs further validation [64].

A summary of the current use of classical serum tumor markers is displayed in Table 1.

## 3. MicroRNAs

### 3.1. Focus on Diagnosis

Of all attempts to find accurate and clinically useful liquid biopsy biomarkers, none has been more remarkable than microRNAs. There is strong evidence that these non-coding RNAs are involved in several aspects of tumor development and progression, having the ability to function either as oncogenes (“oncomiRs”) or tumor suppressors. Their stability in circulation and relatively low-cost methodologies required for detection has attracted researchers to explore these biomarkers in both diagnostic and follow-up, particularly in cancer. Nonetheless, only a few markers have been validated and implemented in the clinic. This may be due to insufficient discrimination from healthy control subjects because of less impressive results when applied to a larger and heterogeneous population and due to technical reasons, namely the interference of pre-clinical variables compromising the detection of the biomarker, among others.

Concerning TGCTs, the recognition of microRNAs of the 371–373 cluster was remarkable and overcame these limitations. Similar to serum tumor markers, there is a link to developmental biology; these microRNAs are involved in the regulation of embryonic development and detected in fetal gonads [65,66]. Furthermore, initial studies in in vitro (cell lines) and in vivo (mouse) models confirm the specificity of these microRNAs for GCTs [67,68,69]. Voorhoeve et al. first pinpointed miR-372 and miR-373 as oncogenic markers in TGCTs, demonstrating their role in the downregulation of the p53 pathway, allowing tumor growth and proliferation in the presence of wild-type *TP53* [70]. This was a groundbreaking work, and soon after, further studies in TGCT tissue samples have also confirmed microRNAs 371–373 and 302 clusters to be highly specific of this tumor type, with levels remarkably distinct from those found in tissue controls [65,69,71,72,73,74,75,76]. These studies also emphasized the universal role of microRNAs, which are expressed across age groups (pediatric and adult patients) and are able to capture all histological diversity (including the precursor lesion GCNIS), except the more differentiated mature teratoma histology [65,69,71,72,73,74,75,76].

Validation of these findings in liquid biopsies was warranted and several research groups have directed efforts towards testing these microRNAs as non-invasive diagnostic markers [67,77]. The first positive evidence was provided by Murray and co-workers, reporting a case of an infant with elevated levels of all these mentioned microRNAs in serum, with their profile mimicking the disease status during treatment [78]. Importantly, an optimized and reproductible protocol with multiplexed reverse transcription and preamplification followed by singleplex PCR was introduced, and consequently adopted in subsequent studies, with minor adjustments [79]. Subsequent works explored the diagnostic value of these microRNAs in TGCTs, further demonstrating that teratoma was the single histological subtype not detected by this test. In further studies, authors tried to direct their research designs to answer clinical questions, and verified that higher microRNA levels (and with a very appreciable fold-change) were observed in patients with TGCTs (compared to normal healthy male subjects and other non-germ cell malignancies of the testis); levels rapidly decreased after orchiectomy (with a half-life shorter than 24 h) [80]; levels reflected tumor burden (tumor size, disease stage, and IGCCCG category); and the microRNA profile reflected the response to chemotherapy. Importantly, in back-to-back comparison studies, the diagnostic accuracy of these microRNAs, individually or as a whole, surpassed the classical serum tumor markers (Table 2), both in sensitivity and specificity (with one of the first reports showing a sensitivity and specificity of serum miR-371a-3p for TGCT diagnosis of 84.7 and 99%) [74,81,82,83,84,85,86,87].

From the extensive evaluation of the different clusters, it was recognized that miR-371a-3p stood out as the most informative [84,85], and further works have validated and showed that this microRNA alone was, in practice, as useful as the combination with the remainder [86]. Importantly, this microRNA was shown to identify malignant GCTs in the case of equivocal and non-diagnostic imaging findings, demonstrating its practical clinical relevance [88].

At this point already, these microRNAs (and miR-371a-3p in particular) seem to check most of the requirements for a reliable liquid biopsy marker [89]. Nevertheless, two important studies (one in serum and one in plasma), having the strength of being multicentric in nature, were even more determinant to confirm the true application of this marker in the clinical setting. First, the prospective study by Dieckmann and collaborators, including 616 TGCT patients and 258 male controls, disclosed a diagnostic sensitivity, specificity and area under the curve of 90.1%, 94.0% and 0.966, respectively, for miR-371a-3p. Its accuracy clearly surpasses the combined sensitivity of classical serum markers across histologies (seminomas and non-seminomas) and clinical stages (I to III) [90]. Additionally, Nappi and co-workers disclosed 100% specificity and 96% sensitivity for confirming active germ cell malignancy in 111 patients with history of GCT [91]. These large studies were decisive for prompting miR-371a-3p forward and towards implementation in real-life clinical context [92]. Finally, interesting work by Lembeck and collaborators indicate that miR-371a-3p could solve the issue reported above related to non-specific elevations of AFP due to liver cytotoxicity from chemotherapy agents, since miR-371a-3p remained undetectable in this scenario [93].

With the growing excitement around these microRNA biomarkers, ongoing and future studies have included more specific clinical questions in their experimental setting [94]. Some studies have investigated the role of pre-analytical variables in microRNA quantification (related to type of sample, hemolysis, PCR contaminants), compared different isolation protocols and pipelines, and explored issues related to normalization and quality control, mostly concluding that if the proper quality control steps are considered and the assays and procedures are standardized, the common techniques available could provide clinically useful information [67,95,96,97]. Other have demonstrated that these microRNAs could be detected in other bodily fluids apart from blood derivatives (malignant pleural effusions, seminal plasma, hydrocele fluid, cerebral spinal fluid), which gave further information about these biomarkers [67,83,98]. The evidence of stability in other fluids argues in favor of their use, for instance, in a metastatic context and in extragonadal GCTs, which was further confirmed [99,100]. These microRNAs were not detected in urine and studies in seminal plasma disclosed issues hampering the use of this bodily fluid for the diagnosis of TGCTs because of higher levels in the healthy control population compared to those observed in serum [83,101,102,103]. However, these studies have reinforced the evidence of the cellular origin of miR-371a-3p from the germ cell compartment, and not from other urogenital tract locations [104]. Other works have, still, explored further markers. For instance, the microRNAs of the “chromosome-19-microRNA-cluster” (C19MC) were also identified as over-represented in TGCTs, particularly in non-seminomas and more aggressive subtypes [105], being useful for the management of patients with choriocarcinoma [106].

Since the early detection of GCNIS is another clinical issue, namely in surveillance of the contralateral testis, studies have been performed to answer this question, but results were not as strong as for invasive forms of the disease, with levels either not differing from control subjects or being higher than the control population but not showing the same diagnostic power as that of invasive forms of the disease [75,83,86].

### 3.2. Focus on Follow-Up

Similarly, studies have addressed with particular attention low burden clinical stage I disease [90]. Ideally, miR-371a-3p testing in an immediate post-orchiectomy setting in patients with stage I tumors should be sensitive enough to predict if, in the near future, such patients would experience relapse or, oppositely, be cured. This would be of great importance, since it would aid clinicians in one of the most difficult routine decisions, which is to safely propose patients for surveillance alone or introduce adjuvant chemotherapy in young-adult men. While a recent study showed that measurement of this marker by the current real-time quantitative PCR methodology was not able to do so immediately after orchiectomy, miR-371a-3p did detect relapse much better than classical serum markers in this context (94.1% patients with elevated miR-371a-3p compared to elevation of classical markers in only 38% at the same timing) [107]. The study by Bagrodia and collaborators on stage I non-seminomas compared the standard model of assessing patients with one introducing miR-371a-3p and demonstrated the impact of adding this biomarker to follow-up, contributing to avoiding overtreatment or undertreatment in a cost-effective way [108].

Besides pre-orchiectomy diagnosis, these microRNAs were also explored as a means of answering the clinical dilemmas during follow-up, mentioned in the Introduction section. For instance, studies have demonstrated that miR-371a-3p levels were elevated in the event of disease recurrence [90,109] and could identify such relapses with more accuracy and earlier than classical markers [110]. This has clinical utility for the follow-up of patients, including interpretation of the response to chemotherapy and determination of the prognosis of patients undergoing systemic treatment, including progression-free survival [111,112].

### 3.3. Focus on the Identification of Metastatic Viable Disease/Teratoma

Since upfront knowledge about the presence of active non-teratoma disease in retroperitoneum post-chemotherapy is desirable to better adjust treatment strategy (resection, or most likely further chemotherapy if a viable tumor remains), relevant work by Leão et al. showed that miR-371a-3p could be useful in indicating the presence of active GCT components [113], a finding later confirmed by other studies, including in chemo-naïve specimens [67,114]. Curiously, the recent study of Dieckmann and collaborators evidenced detectable levels of miR-371a-3p in the cystic fluid of post-chemotherapy teratoma patients despite normal serum levels, which may indicate that viable non-teratoma components present prior to therapy may have secreted this microRNA into the fluid, which accumulated due to the absence of a specific drainage system [115]. Importantly, and since all studies agreed that these embryonic microRNAs were not detected in appreciable levels in mature teratoma, studies have attempted to uncover a so-called “teratoma marker”. Stemming from evidence at the tissue level indicating miR-375 is over-represented in this specific subtype [116], studies have tested such a marker in liquid biopsies of patients with primary or post-chemotherapy retroperitoneal teratoma disease. Whereas the first studies in serum failed to show the benefit of this marker, mainly because of detection in other histologies and in healthy subjects [67,114,117], the study in plasma by Nappi et al. evidenced the benefit of combining miR-371a-3p and miR-375 in a panel for more accurately performing such discrimination, with an area under the curve of 0.95 [118].

All these data clearly support integration of miR-371a-3p in the clinic as a complement of the currently available diagnosis and follow-up methods [25,119]. A list of some of the most representative works on microRNAs in the TGCT field is presented in Table 3. Future studies could possibly point out other microRNAs or even other classes of non-coding RNAs (still scarcely explored in TGCTs) that are useful in specific scenarios [120,121].

## 4. Cell Free DNA (cfDNA)

Circulating cfDNA is long recognized to be a valuable tool for liquid biopsy testing. It is thought to be released into the bloodstream by apoptosis or necrosis and consists of double-stranded segments of 150 to 200nt in length (which corresponds to nucleosome-associated DNA). However, and despite cancer patients having overall higher cfDNA levels in circulation, accurate techniques need to be employed to separate the specific circulating tumor DNA (ctDNA) from the overall cfDNA, since the proportion of the former within the latter can vary greatly. Nevertheless, a number of downstream applications can be pursued on cfDNA isolated from cancer patients, including the study of mutations, copy number alterations, gene fusions and DNA methylation by use of different techniques such as sequencing-based or PCR-based methods. Analysis of ctDNA may also help surpassing the issue of tumor heterogeneity, which may not be captured by tissue sampling [122,123].

Circulating cfDNA may be an important source of biomarkers with clinical utility in the GCT field. GCTs have higher absolute cfDNA content (both seminomas and non-seminomas) compared to control subjects, discriminating both histologies with 88% sensitivity and 97% specificity (including identification of patients for whom classical serum tumor markers were negative), with levels proportional to the disease stage [124]. Moreover, levels of cfDNA in TGCT patients were shown to be significantly higher in patients with disease progression and also to decrease after chemotherapy. Investigation of these samples allowed for the identification of mutations in *CDC27*, *RBMX*, *TPTE2* and *TSPAN16*, also detected in the primary tumors, although with lower frequency [125]. These results argue in favor of using cfDNA for the follow-up of TGCT patients. In line with this, mutations detected in cerebral spinal fluid ctDNA from patients with central nervous system GCTs were also useful for patient management, with the detection of *KIT* and *NRAS* mutations in 25% of the GCT population [126].

The levels of circulating mitochondrial DNA were additionally found to be increased in the circulations of cancer patients and could be attractive since hundreds of copies are present in each cell, compared to only two for genomic DNA [127]. Actually, these have been shown to be prognostic in other malignancies [128]. In the TGCT field, specifically, circulating mitochondrial DNA was significantly over-represented in cancer patients compared to healthy controls, rendering an area under the curve of 0.787, with 94.3% specificity but only 59.5% sensitivity. Despite no clinical correlates with prognostic variables, a combination of improved techniques for detection and the addition of other markers may be useful, especially in patients with normal AFP and HCG [129].

One of the markers previously reported in TGCTs is hypermethylated *RASSF1A* gene. This tumor suppressor gene, hypermethylated in several cancers (and often part of pan-cancer methylation panels) [130] was also documented as hypermethylated in TGCT tissues and cell lines, including in teratomas, which might complement this gap left by microRNAs [131,132,133]. Moreover, an association with cisplatin resistant phenotype was also disclosed [134]. The single study in liquid biopsies, by the same authors, was based on real-time PCR method performed on cfDNA isolated from serum and treated with a restriction enzyme but detected this marker in only 47% of 73 TGCT patients [135]. However, the marker was not detected in any of the 35 controls included. Authors also disclosed frequent hypermethylation of other gene promoters in serum of TGCT patients, maintaining negativity in the male control population, such as *p14(ARF)*, *PTGS2* and *GSTP1* in 53, 45 and 25% of the samples, respectively. Indeed, combination of the mentioned genes together with *APC* and *p16(INK)* in a panel rendered an area under the curve of 0.834 for diagnosis of TGCTs, with 67% sensitivity and 97% specificity, which was superior to the combination of classical serum tumor markers (58% sensitivity). An increase in sensitivity of the assay could be envisioned to improve the usefulness of these methylation-based biomarkers, and also the use of plasma instead of serum, known to have a greater concentration of tumor-specific cfDNA [136].

Moreover, and given the chromosome constitution of TGCTs with extra X chromosomes, the mechanism of X chromosome inactivation (present physiologically in women) is maintained in TGCT tissues, with activation of the long non-coding RNA *XIST* through demethylation of its promoter. Studies in tissue samples have demonstrated this differential methylation status in *XIST* promoter in TGCTs (demethylated) and healthy males (methylated), which is of diagnostic use [137,138,139]. This has also been explored in the plasma samples of TGCT patients, with Kawakami and collaborators detecting demethylated *XIST* in 64% plasma samples from TGCT patients (71% and 55% seminoma and non-seminoma, respectively), with no detection in the plasma of 24 males with other urological malignancies (bladder and renal cancer). At the time, authors have used the conventional PCR technique on bisulfite-treated DNA, so it is to be determined if accuracy can be increased with improved sensitivity of assays and techniques. Additionally, authors have used a cohort of only 25 TGCT plasma samples, requiring further validation in larger cohorts [140].

The active research in cfDNA-directed methodologies and pipelines will no doubt increase the possibility of finding relevant biomarkers with enough sensitivity to be translated into the clinics, especially in a multi-gene panel approach. Specific challenges to these biomarkers currently being addressed are related to the very low amounts of ctDNA in bodily fluids and its rapid clearance from circulation.

## 5. CTCs

CTCs are amenable to be detected in circulation and portray important “live” information about tumor progression and dissemination. Although recognized for several years, their use in TGCTs has been scarcely explored to date. The first data supporting the presence of CTCs in TGCT patients derive from the detection of HCG-mRNA in apheresis products prior to high-dose chemotherapy, which was more frequent in patients with relapse and with visceral metastases [141]. Similar studies followed, using both germ cell alkaline phosphatase [142], AFP and HCG [143,144], and combinations of markers (HCG, fibronectin, EGFR, CD44, alkaline phosphatase, human endogenous retrovirus type K and *XIST*) [145]; however, sensitivity for identifying CTCs was rather low (<60%), and not always associating with tumor burden. An improvement in methodologies was clearly required.

Isolation of CTCs and the establishment of the most specific markers for identification was reported in one study using GCT cell lines, using fluorescent staining and also the CellSearch™ system [146]. More recently, a study in TGCT patients (*n* = 143) truly demonstrated the presence of such cells in 17.5% of patients (although this varied according to the methodology employed, being lower—11.5%—when using only the CellSearch™ protocol). As expected, the number of positive patients increased in metastatic disease (41%) and, importantly, among patients with chemo-resistant and relapsed disease, all showed detectable CTCs, indicating that the study of such cells may constitutes informative biological material [147]. Non-seminomas, including yolk sac tumor and teratoma, in particular, rendered more frequently detectable CTCs. Furthermore, CTC detection after treatment in poor prognosis patients correlated with radiological and pathological response, which may guide treatment adjustments [148]. Refinements in the way of isolating and selecting relevant tumor cells will be instrumental to better make use of these as biomarkers. In particular, several methods should be combined in order to capture the remarkable heterogeneity of GCTs [149].

Studies on CTCs in TGCTs will most likely benefit from more advanced and widely available techniques. In particular, single-cell profiling of CTCs will allow to better understand tumor heterogeneity of all GCTs subtypes [150,151]. An important challenge is competing with the currently available biomarkers (such as microRNAs, for instance), which can be easily isolated with low cost and low volume of blood compared to the higher volumes needed for CTCs.

## 6. Future Directions

**Figure 2:**
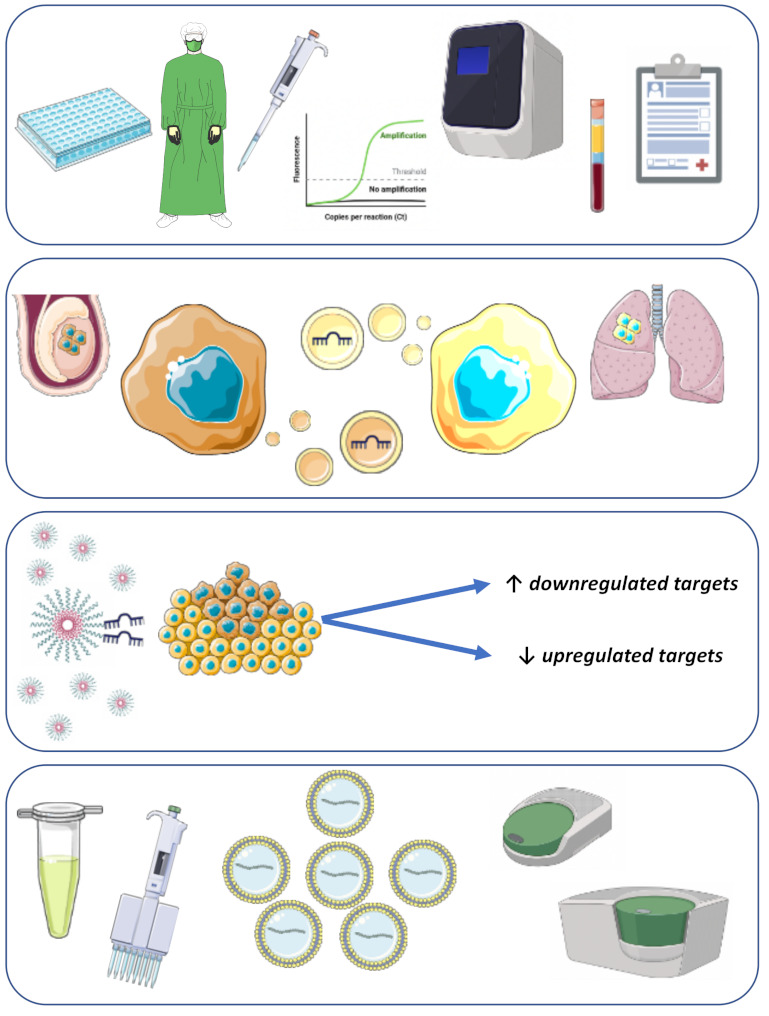
Possible future directions in liquid biopsy testing in TGCTs, including standardization of protocols and reports, studies in exosomes, biomolecule delivery through nanoparticles, and improved techniques such as droplet digital PCR.

There are still several unanswered questions related to liquid biopsy biomarkers in the field of TGCTs. Despite microRNAs being in the front line and assuming the dominant position, even here, there is room for improvement and continuing research (Figure 2).

A more definitive step towards implementation in the clinics is related to the universalization of protocols, quantification and reporting. Given the more advanced position of microRNAs, this might represent one of the final steps before clinical implementation occurs. Here, specifically, and despite results having been widely validated in the most diverse instances and populations, there are variations in pipelines used by different research groups, including isolation techniques, the amount of sample used, the preamplification protocol, normalization (an important issue when working with microRNAs or any other molecular marker), quality control steps (including the evaluation of non-human spike-ins), the type of sample (serum or plasma, or even others), susceptibility to hemolysis and contaminants, and the method of reporting results. A call for the definition of the most robust pipeline has been advocated by other authors, and parallel comparison of protocols will be fundamental to achieve this [94].

One of the evident questions related to the use of microRNAs is linked to extracellular vesicles. This niche in research is rapidly advancing, with improved methodologies for isolating and characterizing such vesicles. These are involved in cell-to-cell communication and processes such as metastatic dissemination and tropism to certain metastatic niches due to the messenger molecules they carry, which include (among others) microRNAs. Importantly, these improvements have ultimately led to refinements in sensitivity and utility drawn from liquid biopsy biomarkers [152]. Although this has been briefly addressed in TGCTs, with the available data not supporting a particular benefit in the isolation protocol from adding an enrichment step for exosomal microRNAs [67], this could definitely be better explored, in particular: the use of more advanced methodologies for isolation (differential ultracentrifugation, size-based and microfluidic-based procedures, or combinations of these); improved characterization of isolated vesicles (nanoparticle tracking analysis-based, fluorescent-activated cell sorting, western blotting or mass-spectrometry-based methods); and exploring of other vesicles besides exosomes, such as microvesicles [153].

In recent years, the idea of using microRNAs to deliver treatment directed at tumor cells has been an active field of research. Indeed, and given the almost universal presence of miR-371a-3p among GCTs, microRNA-based therapies could be envisioned. Two types of strategies could be pursued: both anti-miR, directed at targeting upregulated targets; and therapy based on replenishment of downregulated targets [154]. The latter has been actually demonstrated by Murray et al., with the delivery of a microRNA mimic of let-7 to TGCT cells, which resulted in decreased tumor growth due to the repression of oncogenic mRNA transcripts [155]. Technological advances such as delivery via nanoparticles could be envisioned for this, and motivate studies in this direction, since some microRNA-based therapeutics are already in clinical trials [156,157]. For this, however, more profound knowledge about the pathway interactions of these microRNAs is needed. In fact, and despite the overwhelming data in the practical use of microRNAs of the miR-371-373 cluster, this was not accompanied by the same investment in increasing biological understanding of the functionality of these epigenetic players. Besides the mentioned interaction with the p53 pathway by targeting the tumor suppressor *LATS2*, there are still many more mechanisms to study related to the microRNAs of this cluster. Recently, the finding of the upregulation of miR-885-5p in teratoma and the fact that it promotes the activation of the p53 pathway led to the proposal of a “microRNA switch”, specifically involving miR-371a-3p and miR-885-5p, related to this pathway and non-teratoma/teratoma histologies, respectively [67]. Furthermore, the study of the epigenetic regulation of these microRNAs by promoter methylation could be interesting (since a significant anti-correlation between the expression and methylation was disclosed for miR-373), although better definition of relevant promoter regions and sites needs to be ascertained [66]. Further studies would be instrumental to explore in depth the biology behind these biomarkers, since additional clues could be obtained that enhance its utility in the clinic.

Two particular settings are still in need of advances. First, a robust, very early and reliable marker for risk stratifying stage I TGCT patients is needed, with miR-371a-3p being the best so far but still having room for improvement [107]. Combination with additional markers could be envisioned, but the sensitivity of any specific assay would have to be very high to capture trace levels of the biomarker indicative of future relapse, allowing timely adjustment of strategy, sparing patients unnecessary exposure to chemotherapy or reducing the frequency of CT follow-up scans and hence exposure to radiation. This could have an additional benefit on costs with such imaging procedures and follow-up appointments, as reported in an economic analysis [158]. The current trials for miR-371a-3p will hopefully shed additional light on this issue (NCT03067181, NCT04435756). It can be hypothesized that novel techniques such as droplet digital PCR could increase the sensitivity enough to reliably detect biomarkers predictive of relapse in low burden disease in a timely manner, and this should be explored in the future. The other setting is the one of cisplatin-resistant tumors. Since these patients are responsible for the highest morbidity and mortality related to this disease, there have been various efforts directed towards finding novel targeted therapies for these patients [51,159,160]. Broad characterization of these refractory tumor cells and tissue samples in ongoing and future studies will be instrumental to pinpoint specific markers robust enough to predict the emergence of such resistant phenotypes, so that it can be anticipated and prevented. Furthermore, it will disclose biomarkers of response to alternative treatments (such as epigenetic-based ones) that can be combined with cisplatin and even rescue sensitivity to this drug [161,162,163,164,165,166,167]. In particular, biomarkers of sensitivity to immunotherapies could be envisioned to better select patients for these therapies and improve the results of recent trials [168].

Moreover, studies in proteomics, especially taking advantage of technologies such as mass spectrometry, may bring further novelty to the field [169]. In fact, most proteomic-based studies in this area have focused on tissues instead of liquid biopsies and have disclosed putative biomarkers up- or down-regulated in specific histological subtypes, such as GSTM3, GCP6, CDK10, STARD7, DMTR1, PIWIL1, TMPRSS12 and PAK4, most being implicated in cell cycle regulation, apoptosis, and metabolism [170,171,172,173]. Additionally, studies in representative cell lines may be informative for guiding further work in patient samples [174]. There is an opportunity to apply these methodologies to serum investigations as well, as performed by Strenziok and collaborators [175], who through surface-enhanced laser desorption ionization time-of-flight mass spectrometry (SELDI-TOF MS) disclosed a cluster of proteins differentially present in TGCT patients compared to healthy subjects. In particular, such an approach could also be envisioned for seminal plasma [176], with a recent study already demonstrating distinct proteome of spermatozoa from patients with seminomas and non-seminomas of the testis [177].

Although susceptibility to TGCTs is polygenic in nature, which could hamper efficient population screening [178,179,180], developmental-related parameters and certain mutations can be envisioned as interesting biomarkers of TGCTs in risk populations, including in liquid biopsies [181]. Furthermore, most studies (as evidenced in this review) have been focusing primordially on type II young adult TGCTs, and more work directed towards other GCTs (pediatric, extragonadal, ovarian, etc.) are needed.

To conclude, TGCTs represent an optimal tumor model for research in liquid biopsies. The need for non-invasive ways of diagnosing these tumors, afflicting young patients, will continue to drive research in the field. Serum biomarkers are (and will continue to be) part of the routine management and follow-up of these patients, but there is room for improvement. Complementing the current classical markers with these emerging biomarkers (with the great promise of microRNAs) will provide answers to more specific clinical questions, contributing to personalized and precision medicine. The future is optimistic, with the advent of novel methodologies for improving the sensitivity of detection promising to increase the diagnostic and prognostic biomarker compendium available in the clinics.

## Figures and Tables

**Table 1:** Classical serum tumor markers and their utility in testicular germ cell tumors.

Positivity in Histological Types	AFP	HCG	LDH
GCNIS	-	-	-
Seminoma	<3%	18–31%	29%
Non-seminoma	60–70%	53%	39%
Embryonal carcinoma	40%	25%	20%
Yolk sac tumor	>95%	<5%	10%
Choriocarcinoma	<5%	>95%	20%
Teratoma	20-25%	10%	<5%
**Staging—S stage**	**AFP (µg/L)**	**HCG (IU/L)**	**LDH (U/L)**
S0	<ULN	<ULN	<ULN
S1	<1000	<5000	<1.5× ULN
S2	1000–10,000	5000–50,000	1.5–10× ULN
S3	>10,000	>50,000	>10× ULN
**Prognosis—IGCCCG**	**Seminoma**		**Non-seminoma**
Good	Any location Normal AFP Any HCG or LDH Absence of non-pulmonary visceral metastases		Testicular or retroperitoneal Absence of non-pulmonary visceral metastases AFP < 1000 HCG < 5000 LDH < 1.5× ULN
Intermediate	Any location Normal AFP Any HCG or LDH Presence of non-pulmonary visceral metastases		Testicular or retroperitoneal Absence of non-pulmonary visceral metastases AFP 1000–10,000 HCG 5000–50,000 LDH 1.5×–10× ULN
Poor	-		All mediastinal tumors; testicular or retroperitoneal with any of the features below: Presence of non-pulmonary visceral metastases AFP > 10,000 HCG > 50,000 LDH > 10× ULN

Abbreviations: GCNIS—germ cell neoplasia in situ; ULN—upper limit normal.

**Table 2:** Diagnostic performance of classical serum tumor markers versus microRNAs in liquid biopsy testing (adapted from [25]).

Diagnostic Performance					
Parameter	AFP	HCG	miR-371a-3p	miR-373-3p	miR-367-3p
Positive Predictive Value (%)	32	42	97	94	92
Negative Predictive Value (%)	78	76	83	58	63
AUC (%)	57	69	97	77	86

Abbreviations: AUC—area under the curve.

**Table 3:** Some studies on microRNAs in the field of germ cell tumors, according to design and main context.

Focus on Diagnosis			
Study	Population	Performance	Reference
Gillis et al., 2013	80 GCT 47 controls 12 non-GCT	Sensitivity: 98% Specificity: 48.3%	[82]
Syring et al., 2015	Discovery cohort: 30 TGCT and 18 controls Validation cohort: 59 TGCT, 17 non-GCT and 84 controls	Sensitivity: 84.7% Specificity: 99.0%	[84]
van Agthoven et al., 2017	250 GCT 104 controls 60 non-GCT	Sensitivity: 90.0% Specificity: 86.0%	[86]
Dieckmann et al., 2017	106 TGCT 106 controls	Sensitivity: 88.7% Specificity: 93.4%	[85]
Dieckmann et al., 2019	616 TGCT 258 controls	Sensitivity: 90.1% Specificity: 94.0%	[90]
Badia et al., 2021	58 TGCT 11 controls	Sensitivity: 93.0% Specificity: 100%	[92]
**Focus on Follow-up**			
**Study**	**Findings**		**Reference**
van Agthoven et al., 2017	Identification of disease relapse, outperforming classical markers		[110]
Terbuch et al., 2018	Confirmation of power to identify patients with refractory disease		[109]
Rosas Plaza et al., 2019	Confirmation of power to identify patients with refractory disease		[111]
Mego et al., 2019	miR-371a-3p levels at start of chemotherapy are of prognostic value; association with progression-free survival		[112]
Nappi et al., 2019	Identification of active germ cell malignancy in 111 patients with history of GCT (Sensitivity: 96.0%, Specificity: 100%)		[91]
Morup et al., 2020	MicroRNAs related to patient trajectories; comparison of distinct methodologies for isolating and quantifying microRNAs		[97]
Bagrodia et al., 2020	miR-371a-3p aids in adjusting treatment, in a cost-effective way		[108]
Lobo et al., 2021	Immediate post-orchiectomy miR-371a-3p levels do not predict relapse of stage I patients put on surveillance, but outperform classical markers during follow-up for identifying relapse		[107]
**Focus on Identification of Metastatic Viable Disease/Teratoma**			
**Study**	**Findings**		**Reference**
Leão et al., 2018	miR-371a-3p predicts viable disease after chemotherapy (AUC = 0.874)		[113]
Lobo et al., 2019	miR-371a-3p confirmed to detect viable disease after chemotherapy		[67]
Lafin et al., 2019	miR-371a-3p predicts viable disease in chemo-naïve context		[114]
Nappi et al., 2020	Combination of miR-371a-3p and miR-375 improves discrimination of teratoma from viable disease		[118]
**Others**			
**Study**	**Findings**		**Reference**
Spiekermann et al., 2015	Detection in seminal plasma, pleural effusion, no detection in urine		[83]
Dieckmann et al., 2016	Detection in testicular vein blood and hydrocele fluid		[98]
Murray et al., 2016	Detection in cerebral spinal fluid of pediatric patients with GCT		[99]
Anheuser et al., 2017	miR-371a-3p levels would have resulted in change of clinical approach to five seminoma patients		[88]
Pelloni et al., 2017	Detection in seminal plasma		[101]
Radtke et al., 2017	Detection of GCNIS in serum		[75]
Radtke et al., 2018	miR-371a-3p has a very short half-life (<12 h)		[80]
Radtke et al., 2019	Detection in seminal plasma, but not different from controls		[103]
Boellaard et al., 2019	Reinforcement of origin of miR-371a-3p from the germ cell compartment		[104]
Spiller and Lobo et al., 2020	Detection in seminal plasma; positive correlation with CRIPTO		[102]
Dieckmann et al., 2020	Detection in cystic fluid of post-chemotherapy teratoma, possibly originating from active germ cell tumor before therapy		[115]
Murray et al., 2020	Detection in cerebral spinal fluid; utility for detection of intracranial GCT		[100]
Belge et al., 2020	miR-371a-3p is not elevated in non-GCT (*n* = 99)		[87]

Abbreviations: AUC—area under the curve; GCNIS—germ cell neoplasia in situ; GCT—germ cell tumor; TGCT—testicular germ cell tumor.

## References

[1] Oosterhuis J.W., Looijenga L.H.J. (2019). Human germ cell tumours from a developmental perspective. Nat. Rev. Cancer.

[2] Lobo J., Gillis A.J.M., Jeronimo C., Henrique R., Looijenga L.H.J. (2019). Human Germ Cell Tumors are Developmental Cancers: Impact of Epigenetics on Pathobiology and Clinic. Int. J. Mol. Sci..

[3] Lobo J., Costa A.L., Vilela-Salgueiro B., Rodrigues A., Guimaraes R., Cantante M., Lopes P., Antunes L., Jerónimo C., Henrique R. (2018). Testicular germ cell tumors: Revisiting a series in light of the new WHO classification and AJCC staging systems, focusing on challenges for pathologists. Hum. Pathol..

[4] Cheng L., Albers P., Berney D.M., Feldman D.R., Daugaard G., Gilligan T., Looijenga L. (2018). Testicular cancer. Nat. Rev. Dis. Primers.

[5] Bagrodia A., Albany C., Masterson T.A. (2018). Germ Cell Tumors: Updates on Epidemiology, Biology, and Treatment Considerations. Adv. Urol..

[6] Rajpert-De Meyts E., McGlynn K.A., Okamoto K., Jewett M.A., Bokemeyer C. (2016). Testicular germ cell tumours. Lancet.

[7] Park J.S., Kim J., Elghiaty A., Ham W.S. (2018). Recent global trends in testicular cancer incidence and mortality. Medicine.

[8] Dieckmann K.P., Frey U., Lock G. (2013). Contemporary diagnostic work-up of testicular germ cell tumours. Nat. Rev. Urol..

[9] Dieckmann K.P., Simonsen-Richter H., Kulejewski M., Anheuser P., Zecha H., Isbarn H., Pichlmeier U. (2019). Serum Tumour Markers in Testicular Germ Cell Tumours: Frequencies of Elevated Levels and Extents of Marker Elevation Are Significantly Associated with Clinical Parameters and with Response to Treatment. BioMed Res. Int..

[10] Dieckmann K.P., Kulejewski M., Heinemann V., Loy V. (2011). Testicular biopsy for early cancer detection—Objectives, technique and controversies. Int. J. Androl..

[11] De Rubis G., Rajeev Krishnan S., Bebawy M. (2019). Liquid Biopsies in Cancer Diagnosis, Monitoring, and Prognosis. Trends Pharmacol. Sci..

[12] Chovanec M., Kalavska K., Mego M., Cheng L. (2020). Liquid biopsy in germ cell tumors: Biology and clinical management. Expert Rev. Mol. Diagn..

[13] Chung P., O’Malley M.E., Jewett M.A.S., Bedard P.L., Panzarella T., Sturgeon J., Moore M.J., Hamilton R., Hansen A.R., Anson-Cartwright L. (2019). Detection of Relapse by Low-dose Computed Tomography During Surveillance in Stage I Testicular Germ Cell Tumours. Eur. Urol. Oncol..

[14] Chovanec M., Hanna N., Cary K.C., Einhorn L., Albany C. (2016). Management of stage I testicular germ cell tumours. Nat. Rev. Urol..

[15] Leao R., Ahmad A.E., Hamilton R.J. (2019). Testicular Cancer Biomarkers: A Role for Precision Medicine in Testicular Cancer. Clin. Genitourin. Cancer.

[16] Chovanec M., Abu Zaid M., Hanna N., El-Kouri N., Einhorn L.H., Albany C. (2017). Long-term toxicity of cisplatin in germ-cell tumor survivors. Ann. Oncol..

[17] Fung C., Dinh P., Ardeshir-Rouhani-Fard S., Schaffer K., Fossa S.D., Travis L.B. (2018). Toxicities Associated with Cisplatin-Based Chemotherapy and Radiotherapy in Long-Term Testicular Cancer Survivors. Adv. Urol..

[18] Lago-Hernandez C.A., Feldman H., O’Donnell E., Mahal B.A., Perez V., Howard S., Rosenthal M., Cheng S.C., Nguyen P.L., Beard C. (2015). A refined risk stratification scheme for clinical stage 1 NSGCT based on evaluation of both embryonal predominance and lymphovascular invasion. Ann. Oncol..

[19] Lobo J., Stoop H., Gillis A.J.M., Looijenga L.H.J., Oosterhuis W. (2019). Interobserver Agreement in Vascular Invasion Scoring and the Added Value of Immunohistochemistry for Vascular Markers to Predict Disease Relapse in Stage I Testicular Nonseminomas. Am. J. Surg. Pathol..

[20] Boormans J.L., Mayor de Castro J., Marconi L., Yuan Y., Laguna Pes M.P., Bokemeyer C., Nicolai N., Algaba F., Oldenburg J., Albers P. (2018). Testicular Tumour Size and Rete Testis Invasion as Prognostic Factors for the Risk of Relapse of Clinical Stage I Seminoma Testis Patients Under Surveillance: A Systematic Review by the Testicular Cancer Guidelines Panel. Eur. Urol..

[21] Leão R., Nayan M., Punjani N., Jewett M.A.S., Fadaak K., Garisto J., Lewin J., Atenafu E.G., Sweet J., Anson-Cartwright L. (2018). A New Model to Predict Benign Histology in Residual Retroperitoneal Masses After Chemotherapy in Nonseminoma. Eur. Urol. Focus.

[22] Honecker F., Aparicio J., Berney D., Beyer J., Bokemeyer C., Cathomas R., Cohn-Cedermark G., Daugaard G., Dieckmann K.P., Fizazi K. (2018). ESMO Consensus Conference on testicular germ cell cancer: Diagnosis, treatment and follow-up. Ann. Oncol..

[23] Murray M.J., Huddart R.A., Coleman N. (2016). The present and future of serum diagnostic tests for testicular germ cell tumours. Nat. Rev. Urol..

[24] Milose J.C., Filson C.P., Weizer A.Z., Hafez K.S., Montgomery J.S. (2011). Role of biochemical markers in testicular cancer: Diagnosis, staging, and surveillance. Open Access J. Urol..

[25] Almstrup K., Lobo J., Morup N., Belge G., Rajpert-De Meyts E., Looijenga L.H.J., Dieckmann K.P. (2020). Application of miRNAs in the diagnosis and monitoring of testicular germ cell tumours. Nat. Rev. Urol..

[26] Bergstrand C.G., Czar B. (1956). Demonstration of a new protein fraction in serum from the human fetus. Scand. J. Clin. Lab. Investig..

[27] Gitlin D., Boesman M. (1967). Sites of serum alpha-fetoprotein synthesis in the human and in the rat. J. Clin. Investig..

[28] Abelev G.I., Assecritova I.V., Kraevsky N.A., Perova S.D., Perevodchikova N.I. (1967). Embryonal serum alpha-globulin in cancer patients: Diagnostic value. Int. J. Cancer.

[29] Talerman A., van der Pompe W.B., Haije W.G., Baggerman L., Boekestein-Tjahjadi H.M. (1977). Alpha-foetoprotein and carcinoembryonic antigen in germ cell neoplasms. Br. J. Cancer.

[30] Germa-Lluch J.R., Garcia del Muro X., Maroto P., Paz-Ares L., Arranz J.A., Guma J., Alba E., Sastre J., Aparicio J., Fernández A. (2002). Clinical pattern and therapeutic results achieved in 1490 patients with germ-cell tumours of the testis: The experience of the Spanish Germ-Cell Cancer Group (GG). Eur. Urol..

[31] Blohm M.E., Vesterling-Horner D., Calaminus G., Gobel U. (1998). Alpha 1-fetoprotein (AFP) reference values in infants up to 2 years of age. Pediatr. Hematol. Oncol..

[32] Kitada M., Ozawa K., Sato K., Matsuda Y., Hayashi S., Tokusashi Y., Miyokawa N., Sasajima T. (2011). Alpha-fetoprotein-producing primary lung carcinoma: A case report. World J. Surg. Oncol..

[33] Kawamoto S., Hiraoka T., Kanemitsu K., Kimura M., Miyauchi Y., Takeya M. (1992). Alpha-fetoprotein-producing pancreatic cancer--a case report and review of 28 cases. Hepatogastroenterology.

[34] Murakami T., Yao T., Mitomi H., Morimoto T., Ueyama H., Matsumoto K., Saito T., Osada T., Nagahara A., Watanabe S. (2016). Clinicopathologic and immunohistochemical characteristics of gastric adenocarcinoma with enteroblastic differentiation: A study of 29 cases. Gastric Cancer.

[35] Germa J.R., Llanos M., Tabernero J.M., Mora J. (1993). False elevations of alpha-fetoprotein associated with liver dysfunction in germ cell tumors. Cancer.

[36] Houwert A.C., Giltay J.C., Lentjes E.G., Lock M.T. (2010). Hereditary persistence of alpha-fetoprotein (HPAF P): Review of the literature. Neth. J. Med..

[37] Cole L.A. (2012). The hCG assay or pregnancy test. Clin. Chem. Lab. Med..

[38] Rao C.V., Lei Z.M. (2007). The past, present and future of nongonadal LH/hCG actions in reproductive biology and medicine. Mol. Cell Endocrinol..

[39] Weissbach L., Bussar-Maatz R., Lohrs U., Schubert G.E., Mann K., Hartmann M., Dieckmann K.P., Fassbinder J. (1999). Prognostic factors in seminomas with special respect to HCG: Results of a prospective multicenter study. Seminoma Study Group. Eur. Urol..

[40] Stenman U.H., Alfthan H., Hotakainen K. (2004). Human chorionic gonadotropin in cancer. Clin. Biochem..

[41] Trojan A., Joller-Jemelka H., Stahel R.A., Jacky E., Hersberger M. (2004). False-positive human serum chorionic gonadotropin in a patient with a history of germ cell cancer. Oncology.

[42] Germa J.R., Arcusa A., Casamitjana R. (1987). False elevations of human chorionic gonadotropin associated to iatrogenic hypogonadism in gonadal germ cell tumors. Cancer.

[43] Braunstein G.D., Thompson R., Gross S., Soares J.R. (1985). Marijuana use does not spuriously elevate serum human chorionic gonadotropin levels. Urology.

[44] Jialal I., Sokoll L.J. (2015). Clinical utility of lactate dehydrogenase: A historical perspective. Am. J. Clin. Pathol..

[45] Rampello E., Fricia T., Malaguarnera M. (2006). The management of tumor lysis syndrome. Nat. Clin. Pract. Oncol..

[46] Khan A.A., Allemailem K.S., Alhumaydhi F.A., Gowder S.J.T., Rahmani A.H. (2020). The Biochemical and Clinical Perspectives of Lactate Dehydrogenase: An Enzyme of Active Metabolism. Endocr. Metab. Immune Disord. Drug Targets.

[47] Von Eyben F.E., de Graaff W.E., Marrink J., Blaabjerg O., Sleijfer D.T., Koops H.S., Oosterhuis J.W., Petersen P.H., Echten-Arends J., Jong B. (1992). Serum lactate dehydrogenase isoenzyme 1 activity in patients with testicular germ cell tumors correlates with the total number of copies of the short arm of chromosome 12 in the tumor. Mol. Gen. Genet..

[48] Salem M., Gilligan T. (2011). Serum tumor markers and their utilization in the management of germ-cell tumors in adult males. Expert Rev. Anticancer Ther..

[49] Gilligan T.D., Seidenfeld J., Basch E.M., Einhorn L.H., Fancher T., Smith D.C., Stephenson A.J., Vaughn D.J., Cosby R., Hayes D.F. (2010). American Society of Clinical Oncology Clinical Practice Guideline on uses of serum tumor markers in adult males with germ cell tumors. J. Clin. Oncol..

[50] Jacobsen C., Honecker F. (2015). Cisplatin resistance in germ cell tumours: Models and mechanisms. Andrology.

[51] Oing C., Seidel C., Bokemeyer C. (2018). Therapeutic approaches for refractory germ cell cancer. Expert Rev. Anticancer Ther..

[52] Kollmannsberger C., Tandstad T., Bedard P.L., Cohn-Cedermark G., Chung P.W., Jewett M.A., Powles T., Warde P.R., Daneshmand S., Protheroe A. (2015). Patterns of relapse in patients with clinical stage I testicular cancer managed with active surveillance. J. Clin. Oncol..

[53] Kobayashi K., Saito T., Kitamura Y., Nobushita T., Kawasaki T., Hara N., Takahashi K. (2013). Oncological outcomes in patients with stage I testicular seminoma and nonseminoma: Pathological risk factors for relapse and feasibility of surveillance after orchiectomy. Diagn. Pathol..

[54] Ackers C., Rustin G.J. (2006). Lactate dehydrogenase is not a useful marker for relapse in patients on surveillance for stage I germ cell tumours. Br. J. Cancer.

[55] Fankhauser C.D., Gerke T.A., Roth L., Sander S., Grossmann N.C., Kranzbuhler B., Eberli D., Sulser T., Beyer J., Hermanns T. (2019). Pre-orchiectomy tumor marker levels should not be used for International Germ Cell Consensus Classification (IGCCCG) risk group assignment. J. Cancer Res. Clin. Oncol..

[56] Wilkinson P.M., Read G. (1997). International Germ Cell Consensus Classification: A prognostic factor-based staging system for metastatic germ cell cancers. International Germ Cell Cancer Collaborative Group. J. Clin. Oncol..

[57] Neumann A., Keller T., Jocham D., Doehn C. (2011). Human placental alkaline phosphatase (hPLAP) is the most frequently elevated serum marker in testicular cancer. Aktuelle Urologie.

[58] Koshida K., Uchibayashi T., Yamamoto H., Hirano K. (1996). Significance of placental alkaline phosphatase (PLAP) in the monitoring of patients with seminoma. Br. J. Urol..

[59] De Broe M.E., Pollet D.E. (1988). Multicenter evaluation of human placental alkaline phosphatase as a possible tumor-associated antigen in serum. Clin. Chem..

[60] Lajer H., Daugaard G., Andersson A.M., Skakkebaek N.E. (2002). Clinical use of serum TRA-1–60 as tumor marker in patients with germ cell cancer. Int. J. Cancer.

[61] Gels M.E., Marrink J., Visser P., Sleijfer D.T., Droste J.H., Hoekstra H.J., Andrews P.W., Koops H.S. (1997). Importance of a new tumor marker TRA-1–60 in the follow-up of patients with clinical stage I nonseminomatous testicular germ cell tumors. Ann. Surg. Oncol..

[62] Fossa S.D., Klepp O., Paus E. (1992). Neuron-specific enolase—A serum tumour marker in seminoma?. Br. J. Cancer.

[63] Tandstad T., Klepp O. (2003). Neuron-specific enolase in testicular cancer--clinical experiences with serum neuron-specific enolase in patients with testicular cancer at diagnosis and during follow-up. Acta Oncol..

[64] Narita T., Hatakeyama S., Yoneyama T., Narita S., Yamashita S., Mitsuzuka K., Sakurai T., Kawamura S., Tochigi T., Takahashi I. (2017). Clinical implications of serum N-glycan profiling as a diagnostic and prognostic biomarker in germ-cell tumors. Cancer Med..

[65] Novotny G.W., Belling K.C., Bramsen J.B., Nielsen J.E., Bork-Jensen J., Almstrup K., Sonne S.B., Kjems J., Rajpert-De-Meyts E., Leffers H. (2012). MicroRNA expression profiling of carcinoma in situ cells of the testis. Endocr. Relat. Cancer.

[66] Eini R., Dorssers L.C., Looijenga L.H. (2013). Role of stem cell proteins and microRNAs in embryogenesis and germ cell cancer. Int. J. Dev. Biol..

[67] Lobo J., Gillis A.J.M., van den Berg A., Dorssers L.C.J., Belge G., Dieckmann K.P., Roest H.P., van der Laan L.J.W., Gietema J., Hamilton R.J. (2019). Identification and Validation Model for Informative Liquid Biopsy-Based microRNA Biomarkers: Insights from Germ Cell Tumor In Vitro, In Vivo and Patient-Derived Data. Cells.

[68] Salvatori D.C.F., Dorssers L.C.J., Gillis A.J.M., Perretta G., van Agthoven T., Gomes Fernandes M., Stoop H., Prins J.B., Oosterhuis J.W., Mummery C. (2018). The MicroRNA-371 Family as Plasma Biomarkers for Monitoring Undifferentiated and Potentially Malignant Human Pluripotent Stem Cells in Teratoma Assays. Stem Cell Rep..

[69] Palmer R.D., Murray M.J., Saini H.K., van Dongen S., Abreu-Goodger C., Muralidhar B., Pett M.R., Thornton C.M., Nicholson J.C., Enright A.J. (2010). Malignant germ cell tumors display common microRNA profiles resulting in global changes in expression of messenger RNA targets. Cancer Res..

[70] Voorhoeve P.M., le Sage C., Schrier M., Gillis A.J., Stoop H., Nagel R., Liu Y.P., Duijse J., Drost J., Griekspoor A. (2006). A genetic screen implicates miRNA-372 and miRNA-373 as oncogenes in testicular germ cell tumors. Cell.

[71] Gillis A.J., Stoop H.J., Hersmus R., Oosterhuis J.W., Sun Y., Chen C., Guenther S., Sherlock J., Veltman I., Baeten J. (2007). High-throughput microRNAome analysis in human germ cell tumours. J. Pathol..

[72] Vilela-Salgueiro B., Barros-Silva D., Lobo J., Costa A.L., Guimaraes R., Cantante M., Lopes P., Braga I., Oliveira J., Henrique R. (2018). Germ cell tumour subtypes display differential expression of microRNA371a-3p. Philos. Trans. R. Soc. Lond. B Biol. Sci..

[73] Bing Z., Master S.R., Tobias J.W., Baldwin D.A., Xu X.W., Tomaszewski J.E. (2012). MicroRNA expression profiles of seminoma from paraffin-embedded formalin-fixed tissue. Virchows Arch..

[74] Dieckmann K.P., Spiekermann M., Balks T., Flor I., Loning T., Bullerdiek J., Belge G. (2012). MicroRNAs miR-371–3 in serum as diagnostic tools in the management of testicular germ cell tumours. Br. J. Cancer.

[75] Radtke A., Cremers J.F., Kliesch S., Riek S., Junker K., Mohamed S.A., Anheuser P., Belge G., Dieckmann K.P. (2017). Can germ cell neoplasia in situ be diagnosed by measuring serum levels of microRNA371a-3p?. J. Cancer Res. Clin. Oncol..

[76] Belge G., Hennig F., Dumlupinar C., Grobelny F., Junker K., Radtke A., Dieckmann K.P. (2020). Graded expression of microRNA-371a-3p in tumor tissues, contralateral testes, and in serum of patients with testicular germ cell tumor. Oncotarget.

[77] Rijlaarsdam M.A., van Agthoven T., Gillis A.J., Patel S., Hayashibara K., Lee K.Y., Looijenga L.H.J. (2015). Identification of known and novel germ cell cancer-specific (embryonic) miRs in serum by high-throughput profiling. Andrology.

[78] Murray M.J., Halsall D.J., Hook C.E., Williams D.M., Nicholson J.C., Coleman N. (2011). Identification of microRNAs from the miR-371~373 and miR-302 clusters as potential serum biomarkers of malignant germ cell tumors. Am. J. Clin. Pathol..

[79] Murray M.J., Scarpini C.G., Coleman N. (2021). A Circulating MicroRNA Panel for Malignant Germ Cell Tumor Diagnosis and Monitoring. Methods Mol. Biol..

[80] Radtke A., Hennig F., Ikogho R., Hammel J., Anheuser P., Wulfing C., Belge G., Dieckmann K.P. (2018). The Novel Biomarker of Germ Cell Tumours, Micro-RNA-371a-3p, Has a Very Rapid Decay in Patients with Clinical Stage 1. Urol. Int..

[81] Belge G., Dieckmann K.P., Spiekermann M., Balks T., Bullerdiek J. (2012). Serum levels of microRNAs miR-371–3: A novel class of serum biomarkers for testicular germ cell tumors?. Eur. Urol..

[82] Gillis A.J., Rijlaarsdam M.A., Eini R., Dorssers L.C., Biermann K., Murray M.J., Nicholson J.C., Coleman N., Dieckmann K.P., Belge G. (2013). Targeted serum miRNA (TSmiR) test for diagnosis and follow-up of (testicular) germ cell cancer patients: A proof of principle. Mol. Oncol..

[83] Spiekermann M., Belge G., Winter N., Ikogho R., Balks T., Bullerdiek J., Dieckmann K.P. (2015). MicroRNA miR-371a-3p in serum of patients with germ cell tumours: Evaluations for establishing a serum biomarker. Andrology.

[84] Syring I., Bartels J., Holdenrieder S., Kristiansen G., Muller S.C., Ellinger J. (2015). Circulating serum miRNA (miR-367–3p, miR-371a-3p, miR-372–3p and miR-373–3p) as biomarkers in patients with testicular germ cell cancer. J. Urol..

[85] Dieckmann K.P., Radtke A., Spiekermann M., Balks T., Matthies C., Becker P., Ruf C., Oing C., Oechsle K., Bokemeyer C. (2017). Serum Levels of MicroRNA miR-371a-3p: A Sensitive and Specific New Biomarker for Germ Cell Tumours. Eur. Urol..

[86] Van Agthoven T., Looijenga L.H.J. (2017). Accurate primary germ cell cancer diagnosis using serum based microRNA detection (ampTSmiR test). Oncotarget.

[87] Belge G., Grobelny F., Radtke A., Bodes J., Matthies C., Wulfing C., Dieckmann K.P. (2020). Serum levels of microRNA-371a-3p are not elevated in testicular tumours of non-germ cell origin. J. Cancer Res. Clin. Oncol..

[88] Anheuser P., Radtke A., Wulfing C., Kranz J., Belge G., Dieckmann K.P. (2017). Serum Levels of MicroRNA371a-3p: A Highly Sensitive Tool for Diagnosing and Staging Testicular Germ Cell Tumours: A Clinical Case Series. Urol. Int..

[89] Murray M.J., Coleman N. (2012). Testicular cancer: A new generation of biomarkers for malignant germ cell tumours. Nat. Rev. Urol..

[90] Dieckmann K.P., Radtke A., Geczi L., Matthies C., Anheuser P., Eckardt U., Sommer J., Zengerling F., Trenti E., Pichler R. (2019). Serum Levels of MicroRNA-371a-3p (M371 Test) as a New Biomarker of Testicular Germ Cell Tumors: Results of a Prospective Multicentric Study. J. Clin. Oncol..

[91] Nappi L., Thi M., Lum A., Huntsman D., Eigl B.J., Martin C., O’Neil B., Maughan B.L., Chi K., So A. (2019). Developing a Highly Specific Biomarker for Germ Cell Malignancies: Plasma miR371 Expression Across the Germ Cell Malignancy Spectrum. J. Clin. Oncol..

[92] Badia R.R., Abe D., Wong D., Singla N., Savelyeva A., Chertack N., Woldu S.L., Lotan Y., Mauck R., Ouyang D. (2021). Real-World Application of Pre-Orchiectomy miR-371a-3p Test in Testicular Germ Cell Tumor Management. J. Urol..

[93] Lembeck A.L., Puchas P., Hutterer G., Barth D.A., Terbuch A., Bauernhofer T., Pichler M. (2020). MicroRNAs as Appropriate Discriminators in Non-Specific Alpha-Fetoprotein (AFP) Elevation in Testicular Germ Cell Tumor Patients. Noncoding RNA.

[94] Nappi L., Nichols C. (2019). MicroRNAs as Biomarkers for Germ Cell Tumors. Urol. Clin. N. Am..

[95] Spiekermann M., Dieckmann K.P., Balks T., Bullerdiek J., Belge G. (2015). Is relative quantification dispensable for the measurement of microRNAs as serum biomarkers in germ cell tumors?. Anticancer Res..

[96] Myklebust M.P., Rosenlund B., Gjengsto P., Bercea B.S., Karlsdottir A., Brydoy M., Dahl O. (2019). Quantitative PCR Measurement of miR-371a-3p and miR-372-p Is Influenced by Hemolysis. Front. Genet..

[97] Morup N., Rajpert-De Meyts E., Juul A., Daugaard G., Almstrup K. (2020). Evaluation of Circulating miRNA Biomarkers of Testicular Germ Cell Tumors during Therapy and Follow-up-A Copenhagen Experience. Cancers.

[98] Dieckmann K.P., Spiekermann M., Balks T., Ikogho R., Anheuser P., Wosniok W., Loening T., Bullerdiek J., Belge G. (2016). MicroRNA miR-371a-3p—A Novel Serum Biomarker of Testicular Germ Cell Tumors: Evidence for Specificity from Measurements in Testicular Vein Blood and in Neoplastic Hydrocele Fluid. Urol. Int..

[99] Murray M.J., Bell E., Raby K.L., Rijlaarsdam M.A., Gillis A.J., Looijenga L.H., Brown H., Destenaves B., Nicholson J.C., Coleman N. (2016). A pipeline to quantify serum and cerebrospinal fluid microRNAs for diagnosis and detection of relapse in paediatric malignant germ-cell tumours. Br. J. Cancer.

[100] Murray M.J., Ajithkumar T., Harris F., Williams R.M., Jalloh I., Cross J., Ronghe M., Ward D., Scarpini C.G., Nicholson J.C. (2020). Clinical utility of circulating miR-371a-3p for the management of patients with intracranial malignant germ cell tumors. Neurooncol. Adv..

[101] Pelloni M., Coltrinari G., Paoli D., Pallotti F., Lombardo F., Lenzi A., Gandini L. (2017). Differential expression of miRNAs in the seminal plasma and serum of testicular cancer patients. Endocrine.

[102] Spiller C.M., Lobo J., Boellaard W.P.A., Gillis A.J.M., Bowles J., Looijenga L.H.J. (2020). CRIPTO and miR-371a-3p Are Serum Biomarkers of Testicular Germ Cell Tumors and Are Detected in Seminal Plasma from Azoospermic Males. Cancers.

[103] Radtke A., Dieckmann K.P., Grobelny F., Salzbrunn A., Oing C., Schulze W., Belge G. (2019). Expression of miRNA-371a-3p in seminal plasma and ejaculate is associated with sperm concentration. Andrology.

[104] Boellaard W.P.A., Gillis A.J.M., van Leenders G., Stoop H., van Agthoven T., Dorssers L.C.J., Dinkelman-Smit M., Boormans J.L., Looijenga L.H.J. (2019). Cellular origin of microRNA-371a-3p in healthy males based on systematic urogenital tract tissue evaluation. Andrology.

[105] Flor I., Spiekermann M., Loning T., Dieckmann K.P., Belge G., Bullerdiek J. (2016). Expression of microRNAs of C19MC in Different Histological Types of Testicular Germ Cell Tumour. Cancer Genom. Proteom..

[106] Murray M.J., Smith S., Ward D., Verduci L., Nicholson J.C., Scarpini C.G., Coleman N. (2020). Circulating microRNAs as biomarkers to assist the management of the malignant germ-cell-tumour subtype choriocarcinoma. Transl. Oncol..

[107] Lobo J., Leão R., Gillis A.J.M., van den Berg A., Anson-Cartwright L., Atenafu E.G., Kuhathaas K., Chung P., Hansen A., Bedard P.L. (2020). Utility of Serum miR-371a-3p in Predicting Relapse on Surveillance in Patients with Clinical Stage I Testicular Germ Cell Cancer. Eur. Urol. Oncol..

[108] Bagrodia A., Savelyeva A., Lafin J.T., Speir R.W., Chesnut G.T., Frazier A.L., Woldu S.L., Margulis V., Murray M.J., Amatruda J.F. (2020). Impact of circulating microRNA test (miRNA-371a-3p) on appropriateness of treatment and cost outcomes in patients with Stage I non-seminomatous germ cell tumours. BJU Int..

[109] Terbuch A., Adiprasito J.B., Stiegelbauer V., Seles M., Klec C., Pichler G.P., Resel M., Posch F., Lembeck A.L., Szkandera J. (2018). MiR-371a-3p Serum Levels Are Increased in Recurrence of Testicular Germ Cell Tumor Patients. Int. J. Mol. Sci..

[110] Van Agthoven T., Eijkenboom W.M.H., Looijenga L.H.J. (2017). microRNA-371a-3p as informative biomarker for the follow-up of testicular germ cell cancer patients. Cell Oncol..

[111] Rosas Plaza X., van Agthoven T., Meijer C., van Vugt M., de Jong S., Gietema J.A., Looijenga L.H.J. (2019). miR-371a-3p, miR-373–3p and miR-367–3p as Serum Biomarkers in Metastatic Testicular Germ Cell Cancers Before, During and After Chemotherapy. Cells.

[112] Mego M., van Agthoven T., Gronesova P., Chovanec M., Miskovska V., Mardiak J., Looijenga L.H.J. (2019). Clinical utility of plasma miR-371a-3p in germ cell tumors. J. Cell Mol. Med..

[113] Leão R., van Agthoven T., Figueiredo A., Jewett M.A.S., Fadaak K., Sweet J., Ahmad A.E., Anson-Cartwright L., Chung P., Hansen A. (2018). Serum miRNA Predicts Viable Disease after Chemotherapy in Patients with Testicular Nonseminoma Germ Cell Tumor. J. Urol..

[114] Lafin J.T., Singla N., Woldu S.L., Lotan Y., Lewis C.M., Majmudar K., Savelyeva A., Kapur P., Margulis V., Strand D.W. (2020). Serum MicroRNA-371a-3p Levels Predict Viable Germ Cell Tumor in Chemotherapy-naive Patients Undergoing Retroperitoneal Lymph Node Dissection. Eur. Urol..

[115] Dieckmann K.P., Hennig F., Anheuser P., Gehrckens R., Viehweger F., Wulfing C., Belge G. (2020). High Expression of microRNA-371a-3p in Cystic Fluid of Post-Chemotherapy Teratoma with Concurrent Normal Serum Levels in Patients with Non-Seminomatous Testicular Germ Cell Tumours. Urol. Int..

[116] Shen H., Shih J., Hollern D.P., Wang L., Bowlby R., Tickoo S.K., Thorsson V., Mungall A.J., Newton Y., Hedge A.M. (2018). Integrated Molecular Characterization of Testicular Germ Cell Tumors. Cell Rep..

[117] Belge G., Grobelny F., Matthies C., Radtke A., Dieckmann K.P. (2020). Serum Level of microRNA-375–3p Is Not a Reliable Biomarker of Teratoma. In Vivo.

[118] Nappi L., Thi M., Adra N., Hamilton R.J., Leão R., Lavoie J.M., Soleimani M., Eigl B.J., Chi K., Gleave M. (2021). Integrated Expression of Circulating miR375 and miR371 to Identify Teratoma and Active Germ Cell Malignancy Components in Malignant Germ Cell Tumors. Eur. Urol..

[119] Singla N., Lafin J.T., Bagrodia A. (2019). MicroRNAs: Turning the Tide in Testicular Cancer. Eur. Urol..

[120] Regouc M., Belge G., Lorch A., Dieckmann K.P., Pichler M. (2020). Non-Coding microRNAs as Novel Potential Tumor Markers in Testicular Cancer. Cancers.

[121] Costa A.L., Lobo J., Jeronimo C., Henrique R. (2017). The epigenetics of testicular germ cell tumors: Looking for novel disease biomarkers. Epigenomics.

[122] Corcoran R.B., Chabner B.A. (2018). Application of Cell-free DNA Analysis to Cancer Treatment. N. Engl. J. Med..

[123] Schwarzenbach H., Hoon D.S., Pantel K. (2011). Cell-free nucleic acids as biomarkers in cancer patients. Nat. Rev. Cancer.

[124] Ellinger J., Wittkamp V., Albers P., Perabo F.G., Mueller S.C., von Ruecker A., Bastian P.J. (2009). Cell-free circulating DNA: Diagnostic value in patients with testicular germ cell cancer. J. Urol..

[125] Kubala E., Bakardjieva-Mihaylova V., Skvarova-Kramarzova K., Slamova M., Triska P., Donatova Z., Rozsypalova A., Rosova B., Trka J., Buchler T. (2020). The impact of circulating free tumor DNA (cfDNA) in testicular germ cell tumors (TGCT) management. J. Clin. Oncol..

[126] Takayasu T., Shah M., Dono A., Yan Y., Borkar R., Putluri N., Zhu J.J., Hama S., Yamasaki F., Tahara H. (2020). Cerebrospinal fluid ctDNA and metabolites are informative biomarkers for the evaluation of CNS germ cell tumors. Sci. Rep..

[127] Afrifa J., Zhao T., Yu J. (2019). Circulating mitochondria DNA, a non-invasive cancer diagnostic biomarker candidate. Mitochondrion.

[128] Mehra N., Penning M., Maas J., van Daal N., Giles R.H., Voest E.E. (2007). Circulating mitochondrial nucleic acids have prognostic value for survival in patients with advanced prostate cancer. Clin. Cancer Res..

[129] Ellinger J., Albers P., Muller S.C., von Ruecker A., Bastian P.J. (2009). Circulating mitochondrial DNA in the serum of patients with testicular germ cell cancer as a novel noninvasive diagnostic biomarker. BJU Int..

[130] Malpeli G., Innamorati G., Decimo I., Bencivenga M., Nwabo Kamdje A.H., Perris R., Bassi C. (2019). Methylation Dynamics of RASSF1A and Its Impact on Cancer. Cancers.

[131] Costa A.L., Moreira-Barbosa C., Lobo J., Vilela-Salgueiro B., Cantante M., Guimarães R., Lopes P., Braga I., Oliveira J., Antunes L. (2018). DNA methylation profiling as a tool for testicular germ cell tumors subtyping. Epigenomics.

[132] Lind G.E., Skotheim R.I., Fraga M.F., Abeler V.M., Esteller M., Lothe R.A. (2006). Novel epigenetically deregulated genes in testicular cancer include homeobox genes and SCGB3A1 (HIN-1). J. Pathol..

[133] Honorio S., Agathanggelou A., Wernert N., Rothe M., Maher E.R., Latif F. (2003). Frequent epigenetic inactivation of the RASSF1A tumour suppressor gene in testicular tumours and distinct methylation profiles of seminoma and nonseminoma testicular germ cell tumours. Oncogene.

[134] Koul S., McKiernan J.M., Narayan G., Houldsworth J., Bacik J., Dobrzynski D.L., Assaad A.M., Mansukhani M., Reuter V.E., Bosl G.J. (2004). Role of promoter hypermethylation in Cisplatin treatment response of male germ cell tumors. Mol. Cancer.

[135] Ellinger J., Albers P., Perabo F.G., Muller S.C., von Ruecker A., Bastian P.J. (2009). CpG island hypermethylation of cell-free circulating serum DNA in patients with testicular cancer. J. Urol..

[136] El Messaoudi S., Rolet F., Mouliere F., Thierry A.R. (2013). Circulating cell free DNA: Preanalytical considerations. Clin. Chim. Acta.

[137] Lobo J., Nunes S.P., Gillis A.J.M., Barros-Silva D., Miranda-Goncalves V., Berg A.V.D., van den Berg A., Cantante M., Guimarães R., Henrique R. (2019). XIST-Promoter Demethylation as Tissue Biomarker for Testicular Germ Cell Tumors and Spermatogenesis Quality. Cancers.

[138] Looijenga L.H., Gillis A.J., van Gurp R.J., Verkerk A.J., Oosterhuis J.W. (1997). X inactivation in human testicular tumors. XIST expression and androgen receptor methylation status. Am. J. Pathol..

[139] Kawakami T., Okamoto K., Sugihara H., Hattori T., Reeve A.E., Ogawa O., Okada Y. (2003). The roles of supernumerical X chromosomes and XIST expression in testicular germ cell tumors. J. Urol..

[140] Kawakami T., Okamoto K., Ogawa O., Okada Y. (2004). XIST unmethylated DNA fragments in male-derived plasma as a tumour marker for testicular cancer. Lancet.

[141] Fan Y., Einhorn L., Saxman S., Katz B., Abonour R., Cornetta K. (1998). Detection of germ cell tumor cells in apheresis products using polymerase chain reaction. Clin. Cancer Res..

[142] Hildebrandt M.O., Blaser F., Beyer J., Siegert W., Mapara M.Y., Huhn D., Salama A. (1998). Detection of tumor cells in peripheral blood samples from patients with germ cell tumors using immunocytochemical and reverse transcriptase-polymerase chain reaction techniques. Bone Marrow Transplant..

[143] Yuasa T., Yoshiki T., Tanaka T., Isono T., Okada Y. (1999). Detection of circulating testicular cancer cells in peripheral blood. Cancer Lett..

[144] Hautkappe A.L., Lu M., Mueller H., Bex A., Harstrick A., Roggendorf M., Ruebben H. (2000). Detection of germ-cell tumor cells in the peripheral blood by nested reverse transcription-polymerase chain reaction for alpha-fetoprotein-messenger RNA and beta human chorionic gonadotropin-messenger RNA. Cancer Res..

[145] Bokemeyer C., Gillis A.J., Pompe K., Mayer F., Metzner B., Schleucher N., Schleicher J., Pflugrad-Jauch G., Oosterhuis J.W., Kanz L. (2001). Clinical impact of germ cell tumor cells in apheresis products of patients receiving high-dose chemotherapy. J. Clin. Oncol..

[146] Ruf C., Nastaly P., Becker P., Isbarn H., Honecker F., Pantel K., Riethdorf S., Hoeppner D., Fisch M., Wagner W. (2013). Circulating Tumor Cells Can Be Detected in Patients with Testicular Germ Cell Tumors. J. Urol..

[147] Nastaly P., Ruf C., Becker P., Bednarz-Knoll N., Stoupiec M., Kavsur R., Isbarn H., Matthies C., Wagner W., Hoppner D. (2014). Circulating tumor cells in patients with testicular germ cell tumors. Clin. Cancer Res..

[148] Cebotaru C.L., Buiga R., Placintar A.N., Ghilezan N. (2012). P1.16 Detection of Circulating Tumor Cells Could Adjust Therapy in Poor Risk Germ Cell Tumors? A Pilot Study. Ann. Oncol..

[149] Nastaly P., Honecker F., Pantel K., Riethdorf S. (2021). Detection of Circulating Tumor Cells (CTCs) in Patients with Testicular Germ Cell Tumors. Methods Mol. Biol..

[150] Keller L., Pantel K. (2019). Unravelling tumour heterogeneity by single-cell profiling of circulating tumour cells. Nat. Rev. Cancer.

[151] Cebotaru C.L., Olteanu E.D., Antone N.Z., Buiga R., Nagy V. (2016). Circulating tumor cells in germ cell tumors: Are those biomarkers of real prognostic value? A review. Clujul Med..

[152] Xu R., Rai A., Chen M., Suwakulsiri W., Greening D.W., Simpson R.J. (2018). Extracellular vesicles in cancer—Implications for future improvements in cancer care. Nat. Rev. Clin. Oncol..

[153] Doyle L.M., Wang M.Z. (2019). Overview of Extracellular Vesicles, Their Origin, Composition, Purpose, and Methods for Exosome Isolation and Analysis. Cells.

[154] Murray M.J., Coleman N. (2019). MicroRNA Dysregulation in Malignant Germ Cell Tumors: More Than a Biomarker?. J. Clin. Oncol..

[155] Murray M.J., Saini H.K., Siegler C.A., Hanning J.E., Barker E.M., van Dongen S., Ward D.M., Raby K.L., Groves I.J., Scarpini C.G. (2013). LIN28 Expression in malignant germ cell tumors downregulates let-7 and increases oncogene levels. Cancer Res..

[156] Janssen H.L., Reesink H.W., Lawitz E.J., Zeuzem S., Rodriguez-Torres M., Patel K., van der Meer A.J., Patick A.K., Chen A., Zhou Y. (2013). Treatment of HCV infection by targeting microRNA. N. Engl. J. Med..

[157] Shah M.Y., Ferrajoli A., Sood A.K., Lopez-Berestein G., Calin G.A. (2016). microRNA Therapeutics in Cancer—An Emerging Concept. EBioMedicine.

[158] Charytonowicz D., Aubrey H., Bell C., Ferret M., Tsui K., Atfield R., Coleman N., Murray M.J., Wilson E.C.F. (2019). Cost Analysis of Noninvasive Blood-Based MicroRNA Testing Versus CT Scans for Follow-up in Patients with Testicular Germ-Cell Tumors. Clin. Genitourin. Cancer.

[159] Cardoso A.R., Lobo J., Miranda-Goncalves V., Henrique R., Jeronimo C. (2020). Epigenetic alterations as therapeutic targets in Testicular Germ Cell Tumours: Current and future application of ‘epidrugs’. Epigenetics.

[160] Oing C., Skowron M.A., Bokemeyer C., Nettersheim D. (2019). Epigenetic treatment combinations to effectively target cisplatin-resistant germ cell tumors: Past, present, and future considerations. Andrology.

[161] Cheng M.L., Donoghue M.T.A., Audenet F., Wong N.C., Pietzak E.J., Bielski C.M., Isharwal S., Iyer G., Funt S., Bagrodia A. (2020). Germ Cell Tumor Molecular Heterogeneity Revealed Through Analysis of Primary and Metastasis Pairs. JCO Precis. Oncol..

[162] Barrett M.T., Lenkiewicz E., Malasi S., Stanton M., Slack J., Andrews P., Pagliaro L., Bryce A.H. (2019). Clonal analyses of refractory testicular germ cell tumors. PLoS ONE.

[163] Necchi A., Bratslavsky G., Corona R.J., Chung J.H., Millis S.Z., Elvin J.A., Vergilio J.A., Suh J., Ramkissoon S., Severson E. (2020). Genomic Characterization of Testicular Germ Cell Tumors Relapsing After Chemotherapy. Eur. Urol. Focus.

[164] Loveday C., Litchfield K., Proszek P.Z., Cornish A.J., Santo F., Levy M., Macintyre G., Holryod A., Broderick P., Dudakia D. (2020). Genomic landscape of platinum resistant and sensitive testicular cancers. Nat. Commun..

[165] Fazal Z., Singh R., Fang F., Bikorimana E., Baldwin H., Corbet A., Tomlin M., Yerby C., Adra N., Albany C. (2020). Hypermethylation and global remodelling of DNA methylation is associated with acquired cisplatin resistance in testicular germ cell tumours. Epigenetics.

[166] Singh R., Fazal Z., Corbet A.K., Bikorimana E., Rodriguez J.C., Khan E.M., Shahid K., Freemantle S.J., Spinella M.J. (2019). Epigenetic Remodeling through Downregulation of Polycomb Repressive Complex 2 Mediates Chemotherapy Resistance in Testicular Germ Cell Tumors. Cancers.

[167] Albany C., Fazal Z., Singh R., Bikorimana E., Adra N., Hanna N.H., Einhorn L.H., Perkins S.M., Sandusky G.E., Christensen B.C. (2020). A phase 1 study of combined guadecitabine and cisplatin in platinum refractory germ cell cancer. Cancer Med..

[168] Kalavska K., Schmidtova S., Chovanec M., Mego M. (2020). Immunotherapy in Testicular Germ Cell Tumors. Front. Oncol..

[169] Milardi D., Grande G., Vincenzoni F., Pierconti F., Pontecorvi A. (2019). Proteomics for the Identification of Biomarkers in Testicular Cancer-Review. Front. Endocrinol..

[170] Zimmermann U., Junker H., Kramer F., Balabanov S., Kleist B., Kammer W., Nordheim A., Walther R. (2006). Comparative proteomic analysis of neoplastic and non-neoplastic germ cell tissue. Biol. Chem..

[171] Leman E.S., Magheli A., Yong K.M., Netto G., Hinz S., Getzenberg R.H. (2009). Identification of nuclear structural protein alterations associated with seminomas. J. Cell. Biochem..

[172] Liu M., Hu Z., Qi L., Wang J., Zhou T., Guo Y., Zeng Y., Zheng B., Wu Y., Zhang P. (2013). Scanning of novel cancer/testis proteins by human testis proteomic analysis. Proteomics.

[173] Castillo J., Knol J.C., Korver C.M., Piersma S.R., Pham T.V., de Goeij-de Haas R.R., van Pelt A.M.M., Jimenez C.R., Jansen B.J.H. (2019). Human Testis Phosphoproteome Reveals Kinases as Potential Targets in Spermatogenesis and Testicular Cancer. Mol. Cell. Proteom..

[174] Bremmer F., Bohnenberger H., Kuffer S., Oellerich T., Serve H., Urlaub H., Strauss A., Maatoug Y., Behnes C.L., Oing O. (2019). Proteomic Comparison of Malignant Human Germ Cell Tumor Cell Lines. Dis. Markers.

[175] Strenziok R., Hinz S., Wolf C., Conrad T., Krause H., Miller K., Schrader M. (2010). Surface-enhanced laser desorption/ionization time-of-flight mass spectrometry: Serum protein profiling in seminoma patients. World J. Urol..

[176] Milardi D., Grande G., Vincenzoni F., Castagnola M., Marana R. (2013). Proteomics of human seminal plasma: Identification of biomarker candidates for fertility and infertility and the evolution of technology. Mol. Reprod. Dev..

[177] Panner Selvam M.K., Alves M.G., Dias T.R., Pushparaj P.N., Agarwal A. (2020). Distinct Proteomic Profile of Spermatozoa from Men with Seminomatous and Non-Seminomatous Testicular Germ Cell Tumors. Int. J. Mol. Sci..

[178] Litchfield K., Mitchell J.S., Shipley J., Huddart R., Rajpert-De Meyts E., Skakkebaek N.E., Houlston R.S., Turnbull C. (2015). Polygenic susceptibility to testicular cancer: Implications for personalised health care. Br. J. Cancer.

[179] Loveday C., Law P., Litchfield K., Levy M., Holroyd A., Broderick P., Kote-Jarai Z., Dunning A.M., Muir K., Peto J. (2018). Large-scale Analysis Demonstrates Familial Testicular Cancer to have Polygenic Aetiology. Eur. Urol..

[180] Litchfield K., Loveday C., Levy M., Dudakia D., Rapley E., Nsengimana J., Bishop D.T., Reid A., Huddart R., Broderick P. (2018). Large-scale Sequencing of Testicular Germ Cell Tumour (TGCT) Cases Excludes Major TGCT Predisposition Gene. Eur. Urol..

[181] Looijenga L.H.J., Kao C.S., Idrees M.T. (2019). Predicting Gonadal Germ Cell Cancer in People with Disorders of Sex Development; Insights from Developmental Biology. Int. J. Mol. Sci..

